# Severe Hypermagnesemia with Normal Renal Function Can Improve with Symptomatic Treatment

**DOI:** 10.1155/2020/2918249

**Published:** 2020-07-14

**Authors:** Yoshiaki Ishida, Akihiko Tabuchi

**Affiliations:** Emergency and Critical Care Center, Anjo Kosei Hospital, 28 Higashihirokute, Anjo-cho, Anjo, Aichi 446-8602, Japan

## Abstract

Hypermagnesemia is a rare disorder and commonly occurs in patients with renal dysfunction. Supportive therapy for hypermagnesemia consists of administration of high-volume fluids, calcium preparation, diuretics, and, in severe cases, hemodialysis. Few reports have described severe hypermagnesemia patients with normal renal function who improved without hemodialysis. A 56-year-old woman presented with a history of constipation in spite of taking constipation medicine, including MgO. She was brought to our emergency department due to vomiting and diffuse distension of the abdomen. Sudden vomiting, weakness, and lower level of consciousness occurred during examination. Her blood pressure dropped to 77/34 mmHg, and deep tendon reflexes of the limbs disappeared. Abdominal computed tomography showed bowel distension with wall edema, and biochemical testing showed serum Mg at 13.5 mg/dl. She was diagnosed with severe hypermagnesemia associated with intestinal obstruction and administered intravenous loop diuretics and calcium preparation in addition to high volumes of normal saline. As the serum Mg level steadily declined, her level of consciousness returned to usual. This case suggests that severe hypermagnesemia can occur in patients with normal renal function and constipation under MgO. Severe hypermagnesemia with normal renal function can improve with symptomatic treatment without hemodialysis.

## 1. Introduction

Hypermagnesemia is rare and occurs most commonly in patients after ingestion of magnesium oxide (MgO) [1]. Hypermagnesemia is caused mainly by decreased excretion from the kidney, increased absorption in the intestinal tract, or increased Mg intake [2]. Generally, hypermagnesemia occurs in elderly patients, especially with renal or bowel dysfunction [3, 4]. Supportive therapy is the mainstay of the management of hypermagnesemia, involving high-volume fluids, diuretics, calcium preparation, and hemodialysis [5]. Notably, severe hypermagnesemia with renal failure and life-threatening symptoms usually requires hemodialysis [3, 6]. However, few reports have described hypermagnesemia in patients with normal renal function [1, 5, 7–9], and even fewer reports have described severe hypermagnesemia that improved with symptomatic treatment alone [7]. We report a severe hypermagnesemia patient with normal renal function, who was treated without hemodialysis.

## 2. Case Presentation

**Figure 1:**
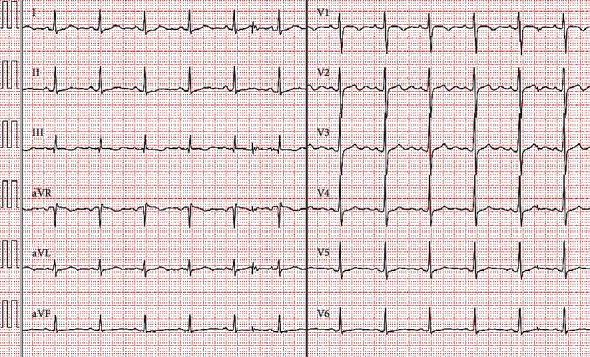
Electrocardiography on arrival in the emergency department demonstrating a prolonged QT interval (QT 442 ms and QTc 531 ms).

**Figure 2:**
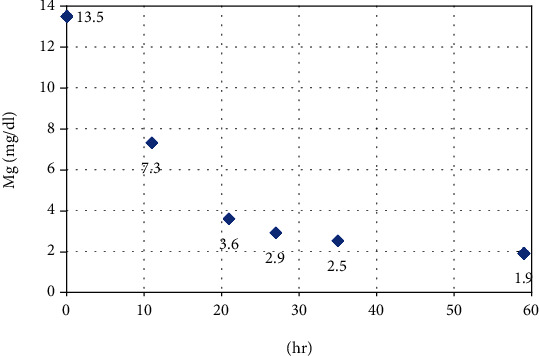
Changes in serum Mg values over time.

A 56-year-old woman presented with an 8-day history of constipation. She had been in a support facility for individuals with disabilities for many years due to Down's syndrome. She was brought to our emergency department (ED) due to vomiting and diffuse distension of the abdomen. She had a history of chronic constipation and had taken pharmacotherapies for constipation, including MgO at 1500 mg/day. Physical examination of the abdomen revealed only nontender distension and tympanic sounds on percussion. Sudden vomiting, weakness, and reduced level of consciousness occurred during abdominal X-ray. While vital signs had been normal on arrival at our ED, her blood pressure dropped to 77/34 mmHg. She also showed a change to absence of deep tendon reflexes of the limbs. Electrocardiography showed a prolonged QT interval (Figure 1), abdominal X-ray showed accumulation of intestinal gas, and abdominal computed tomography showed bowel distension with wall edema. Biochemical testing showed the following: glucose, 135 (73–109) mg/dl; albumin, 3.0 (4.1–5.1) mg/dl; blood urea nitrogen, 18 (8–20) mg/dl; creatinine (Cr), 0.76 (0.46–0.79) mg/dl; estimated glomerular filtration rate (eGFR), 61 ml/min/1.73 m^2^; calcium, 9.2 (8.7–10.3) mg/dl; and Mg, 13.5 (1.8–2.4) mg/dl. From the above, she was diagnosed with severe hypermagnesemia associated with intestinal obstruction. Although we consulted with her family to explore the option of emergency hemodialysis, they instead decided to continue symptomatic treatment. Intravenous loop diuretics and calcium preparation bolus were administered while infusing high volumes of normal saline. She subsequently showed good diuresis and a large amount of defecation the next day. Serum Mg level steadily declined, reaching 7.3 mg/dl after 11 h, 3.6 mg/dl after 21 h, and 2.9 mg/dl after 27 h. By day 3, serum Mg level had almost normalized, at 2.5 mg/dl (Figure 2). As serum Mg level declined, her level of consciousness returned to usual. Eventually, she could walk unaided and was discharged on hospital day 15 with no sequelae.

## 3. Discussion

Hypermagnesemia is a relatively rare disorder and usually is caused by ingestion of MgO [1]. Hypermagnesemia is the least common electrolyte abnormality seen in the ED, accounting for only about 1% of electrolyte abnormality cases [10]. The normal range of serum Mg is 1.7–2.4 mg/dl [2], and symptoms start appearing when Mg concentrations exceed 5 mg/dl. Symptoms include nausea/vomiting, cutaneous flushing, bradycardia, and hypotension [4]. Progression of hypermagnesemia results in the loss of deep tendon reflexes, somnolence, respiratory depression, paralysis, complete heart block, and even cardiac arrest [4] (Table 1). The present patient exhibited vomiting, hypotension, absence of deep tendon reflexes, and somnolence. She was diagnosed with severe hypermagnesemia because her serum Mg concentration (13.5 mg/dl) was at a life-threatening level.

We identified two important clinical issues. First, hypermagnesemia can occur in patients with bowel dysfunction even if renal function is normal and the MgO dose is within the upper limit. Generally, hypermagnesemia occurs in elderly patients, especially those with renal dysfunction or bowel dysfunction [2]. Mg reabsorption and regulation within the kidney principally occur at the epithelial cells of the thick ascending limb of Henle's loop [11]. Henle's loop has the capacity to completely reject Mg reabsorption under conditions of hypermagnesemia, and hence, a maximal renal excretion of more than 6 g/day (500 mEq/day) can occur [4]. Renal dysfunction thus leads to hypermagnesemia, and eGFR <30 ml/min/1.73 m^2^ is also associated with elevated risk of hypermagnesemia [7]. Mg regulation mainly occurs in the small intestine, particularly in the proximal portions [7], and its mechanism of regulation is mainly through passive diffusion [4]. Also, an elevation in Mg concentration is much more likely in the presence of gastrointestinal disorders (active gastric ulcer disease, gastritis, colitis) that can enhance Mg absorption [1]. Therefore, massive Mg ingestion may result in hypermagnesemia if the absorbed amount of Mg goes beyond the renal excretory capacity [4]. However, even if the dosage of oral MgO is within the normal range, hypermagnesemia still can occur depending on complications such as gastrointestinal disorders [1, 4, 7, 8, 12–14]. MgO is often used for chronic constipation, which promotes intestinal absorption of Mg due to long-term contact between the mucous membranes and Mg. When serum Mg levels increase, movement of the intestinal tract is further decreased due to the neuromuscular blocking actions of Mg [7]. As a result, hypermagnesemia worsens. In our case, the patient had no history of renal disease and used MgO appropriately, but developed hypermagnesemia due to chronic constipation.

Our other key finding is that if renal function is normal, severe hypermagnesemia can improve with symptomatic treatment without hemodialysis. Two important supportive therapy principles for hypermagnesemia are removal of Mg from the blood and inhibition of Mg activity [12]. Removal of Mg from the blood can be achieved by high-volume normal saline, loop diuretics, and hemodialysis. Intravenous infusion of normal saline and loop diuretics enhances Mg excretion in patients with normal renal function [3]. In particular, loop diuretics inhibits Mg reabsorption in the thick ascending limb of Henle's loop [8]. Hemodialysis is necessary for patients with renal failure and life-threatening symptoms [3, 6]. Inhibition of Mg activity requires administration of a calcium preparation to reverse cardiac arrhythmia, respiratory depression, and hypotension [3]. Many reports have described the treatment of hypermagnesemia with hemodialysis [1–3, 5–7, 9, 12, 13]. However, few reports have described hypermagnesemia in patients with normal renal function [1, 5, 7–9], and even fewer reports have described severe hypermagnesemia that improved with symptomatic treatment alone [7]. In our case, serum Mg levels in the patient normalized without hemodialysis. Intravenous administration of loop diuretics and calcium preparation while infusing high volumes of normal saline were sufficient to normalize Mg levels in our patient.

We have described a patient with normal renal function who developed severe hypermagnesemia because of chronic constipation and use of MgO. In our case, hypermagnesemia with normal renal function can improve with symptomatic treatment without hemodialysis.

## Figures and Tables

**Table 1:** Symptoms of hypermagnesemia associated with serum Mg levels.

Serum Mg level (mg/dl)	Clinical symptoms
1.7-2.4	Normal serum level
5-8	Nausea/vomiting, cutaneous flushing, bradycardia, hypotension
9-12	Absent deep tendon reflexes, somnolence
>15	Respiratory depression, paralysis, complete heart block
>20	Cardiac arrest
